# Spectrofluorometric and spectrophotometric assay of anticancer drug ensartinib using ion-pair complex formation reaction with eosin Y

**DOI:** 10.1038/s41598-026-64336-8

**Published:** 2026-08-07

**Authors:** Hesham Salem, Nadeen Emad, Hassan Alam, Aya Atef, Malak Galal, Hoda Madian, Dina Z. Mazen, Amany Abdelaziz

**Affiliations:** Pharmaceutical chemistry department, faculty of pharmacy, Deraya University, New Minia, Egypt

**Keywords:** Ensartinib, Eosin Y, Spectrophotometry, Spectrofluorimetry, Pharmaceutical formulations, Biological techniques, Chemistry

## Abstract

Two simple, sensitive, and eco-friendly spectroscopic methods were developed for the determination of ensartinib through ion-pair complexation with eosin Y. Method A represents the first reported spectrofluorometric assay for the studied drug and it is based on fluorescence quenching of eosin Y at pH 3.3, showing linearity over 100–1000 ng mL⁻¹ with LOD and LOQ values of 21.2 and 64.1 ng mL⁻¹, respectively. Method B is a spectrophotometric method based on measuring the absorbance of the formed ion-pair complex at 550 nm, obeying Beer’s law over 1.0–10.0 µg mL⁻¹, with LOD and LOQ values of 132 and 400 ng mL⁻¹, respectively. Both methods were validated according to ICH guidelines and exhibited excellent accuracy (98.51–101.68%) and precision (RSD < 2%). The proposed procedures were successfully applied to capsules, providing mean recoveries of 99.29 ± 1.11% and 100.39 ± 0.98% for Methods A and B, respectively. Statistical comparison with a reported chromatographic method showed no significant difference at the 95% confidence level. The environmental impact of the developed methods was assessed using AGREE and GAPI tools, confirming their compliance with green analytical chemistry principles.

## Introduction

**Fig. 1:**
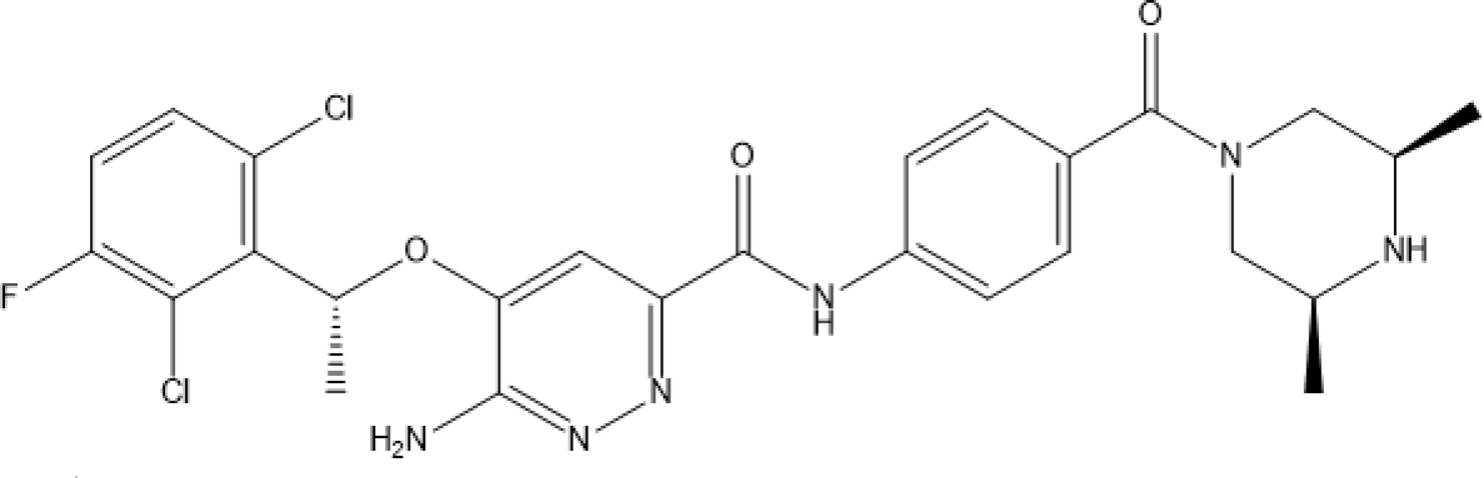
Chemical structure of ENSA.

Ensartinib (ENSA) is a tyrosine kinase inhibitor used in the treatment of ALK‑positive non‑small cell lung cancer^1^. It is 6-amino-5-[(1R)-1-(2,6-dichloro-3-fluorophenyl)ethoxy]-N-[4-[(3R,5 S)-3,5-dimethylpiperazine-1 carbonyl] phenyl] pyridazine-3 carboxamide (Fig. 1). It is an amino pyridazine-based small-molecule tyrosine kinase inhibitor (TKI) that blocks the ALK protein. Compared with crizotinib, ENSA demonstrates approximately ten-fold greater potency in inhibiting ALK-positive lung cancer cell growth^2^. While its therapeutic importance is well established, analytical reports on its determination remain limited, particularly in pharmaceutical dosage forms. Most published methods rely on chromatographic techniques such as LC–MS/MS and UPLC–MS/MS, which have been applied mainly to biological matrices including plasma and liver microsomes. These approaches, although highly sensitive and selective, are constrained by high operational costs, complex sample preparation, and the need for advanced instrumentation and skilled operators. Furthermore, simultaneous multi‑analyte LC–MS/MS assays often compromise analyte‑specific optimization, reducing their suitability for routine dosage‑form analysis^3–6^. Chromatographic techniques, particularly LC–MS/MS and UPLC–MS/MS, have been widely applied to biological matrices such as plasma and liver microsomes, supporting pharmacokinetic and therapeutic drug monitoring studies. These methods provide excellent sensitivity and selectivity but are limited by high cost, complex sample preparation, and the need for sophisticated instrumentation. In contrast, reports on ensartinib determination in pharmaceutical dosage forms remain scarce. To date, no spectrophotometric or spectrofluorometric methods have been reported for the determination of ensartinib in pharmaceutical formulations. Developing such methods is highly desirable, as they offer advantages of simplicity, rapidity, cost‑effectiveness, and environmental compatibility. Eosin Y has been widely employed as an ion‑pairing reagent for the spectroscopic determination of basic pharmaceutical compounds, providing reliable analytical performance with minimal environmental burden^7–10^. In this context, the present study introduces the first spectrophotometric and spectrofluorometric procedures for ensartinib determination in pharmaceutical dosage forms, based on ion‑pair complexation with eosin Y. The novelty of this work lies in the development of simple, rapid, and environmentally friendly procedures; utilizing the (GAPI) green analytical procedure index^11^ and the (AGREE) analytical greenness calculator^12^, based on ion‑pair complexation with eosin Y. This offers high sensitivity and accuracy without the need for sophisticated instrumentation or complex sample preparation. These methods provide a practical alternative to chromatographic techniques and contribute new knowledge to the field of pharmaceutical analysis by extending the applicability of spectroscopic approaches to ensartinib quality‑control testing.

## Experimental

### Instruments

Spectrofluorometric results were carried out using a JASCO FP-8350 Spectrofluorometer equipped with a 150 W xenon arc lamp and a photomultiplier tube operated at 400 V. Both excitation and emission monochromators were scanned at a speed of 1000 nm min⁻¹ with slit widths set to 5 nm. UV-Win software was used to operate a T80 double-beam UV-VIS spectrophotometer (PG Instruments, Leicestershire, UK) for spectrophotometric measurements. Quartz cells with a route length of one centimeter were employed all along. An Aquatron water still 4000d (Cole-Parmer, Staffordshire, UK) was used to create double-distilled water. An analytical balance (Precisa 125 A, Switzerland) was used for all weighing operations.

### Reagents and solvents

Eosin Y was obtained from Merck (Darmstadt, Germany). Eosin Y solutions were prepared at concentrations of 0.5 × 10⁻³ M for the spectrofluorometric study (Method A) and 1.0 × 10⁻⁴ M for the spectrophotometric study (Method B), in accordance with the sensitivity requirements of each procedure. Analytical‑grade dimethylformamide (DMF) was supplied by Fisher Scientific (Loughborough, UK). Methanol, acetonitrile and ethanol were purchased from Merck (Darmstadt, Germany). Acetone, acetic acid, and sodium acetate of analytical grade were obtained from El Nasr Pharmaceutical Chemical Co. (Cairo, Egypt). Although several organic solvents (methanol, ethanol, acetonitrile, acetone, and dimethylformamide) were used during preliminary solvent evaluation during method development, distilled water was selected as the diluting solvent for both methods due to its superior analytical performance and environmental compatibility. Organic solvents were used only where required for initial stock solution preparation to ensure complete dissolution of ensartinib. Acetate buffer solutions (0.1 M) were freshly prepared using appropriate amounts of acetic acid and sodium acetate. Ensartinib (ENSA) raw material (purity ≥ 98%) was kindly supplied by Beijing Sun‑flower Science and Technology Development Co., Ltd. Ensacove^®^ capsules, labeled to contain ensartinib, were used as the commercial pharmaceutical dosage form.

### Calibration solutions preparation

A stock solution of ENSA (100 µg mL⁻¹) was prepared by dissolving 50 mg of the drug in 500 mL of distilled water. This solution was further diluted with distilled water to obtain an intermediate working solution of 20 µg mL⁻¹ for spectrophotometric and spectrofluorometric measurements.

### General procedure

#### Spectrofluorometric method (Method A)

Aliquots of standard ENSA solutions corresponding to concentrations of 100–1000 ng/mL were transferred into 10 mL volumetric flasks. Subsequently, 0.5mL of acetate buffer (0.1 M, pH 3.3) and 1.3 mL of eosin Y solution (5 × 10⁻⁴ M) were added. The solutions were carefully mixed after being diluted to volume with distilled water. After excitation at 350 nm, fluorescence intensities were recorded at 550 nm. The same process was used to create a blank solution without adding ENSA. Plotting the decrease in fluorescence intensity against ENSA concentration allowed for the construction of the calibration curve.

### Spectrophotometric method (Method B)

ENSA calibration solutions (1–10 µg /mL) were produced in 10 mL volumetric flasks, then addition of 0.3 mL buffer of acetate (0.1 M, pH 3.3) and 1.2 mL eosin Y solution (0.1 × 10⁻^4^M). Distilled water was used as a diluting agent, the absorbance at 550 nm was compared to a blank reagent .

### Complex stochiometry

The stoichiometric equilibrium value of ENSA and EY was found by applying the Job’s method of continuous variation^17^. Equimolar solutions (1.3 × 10⁻³ M) were combined in various ratios, buffered with acetate buffer (0.1 M), diluted with re-distilled water, and absorbance was measured at 550 nm. A Job’s plot of adjusted absorbance vs. mole fraction was generated.

### Application of available prescribed forms (capsules)

Twenty Ensacove^®^ capsules were unfilled and blended together. A precise quantity equal to 50 milligrams of ENSA was weighed and put into a 100 mL volumetric flask, where it was dissolved in roughly 50 milliliters of distilled water. The flask’s contents were swirled, 10 min of sonication, and then filled to the volume with distilled water. Following a thoroughly mixing and filtering of the materials, the filtrate’s first part was thrown away. Following the usual analytical procedure, the produced solution was quantitatively further diluted to achieve a final concentration within the calibration’s concentration ranges. of the suggested spectrofluorometric and spectrophotometric procedures.

## Result and discussion

**Fig. 2:**
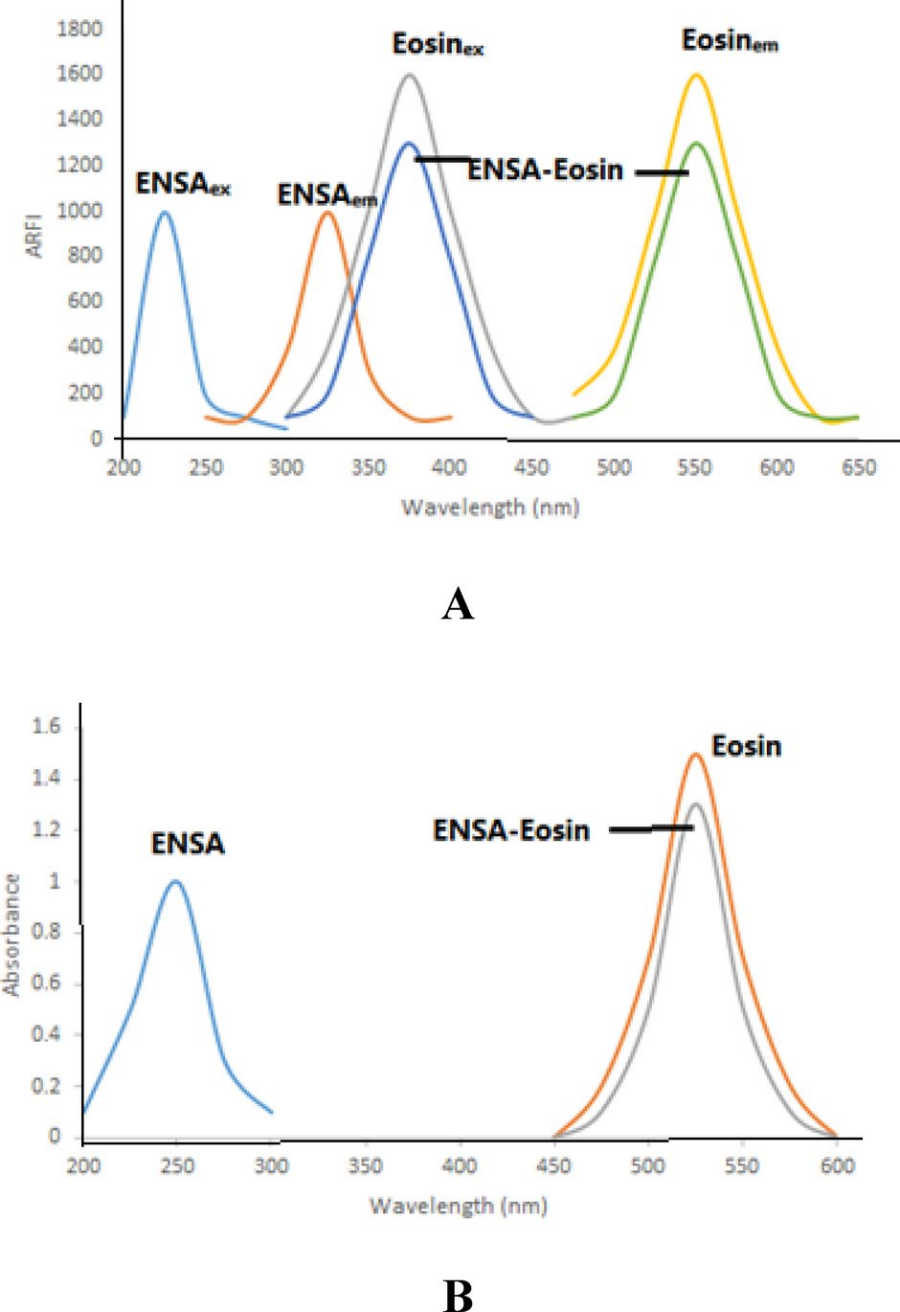
(**A**) Excitation and emission spectra of 300 ng mL^− 1^ ENSA, eosin 5 × 10^− 4^ M, and its reaction with 300 ng mL^− 1^ ENSA. (**B**) Absorbance spectra of 5 µg mL^− 1^ ENSA, eosin, and its reaction with 5 µg mL^− 1^ ENSA, and the insert shows the absorbance spectrum of the complex after subtractingthe eosin blank.

Eosin Y is an acidic xanthene dye that has been widely employed as an ion‑pairing reagent for the spectroscopic determination of basic pharmaceutical compounds. In the present study, under acidic conditions, ensartinib exhibited a concentration‑dependent decrease in the native fluorescence of eosin Y at 550 nm after excitation at 350 nm (Fig. 2A) for method A. A corresponding increase in absorbance at 550 nm (Fig. 2B). The maximum analytical response was achieved in acetate buffer within the pH range of 3.2–3.6, with pH 3.3 selected as optimal. This behavior indicates that acidic conditions are essential for efficient interaction between ensartinib and eosin Y. Stoichiometric investigation using Job’s method revealed a 1:1 molar ratio between ensartinib and eosin Y, supporting the formation of a binary ion‑pair complex. The observed pH dependence, stoichiometric ratio, and characteristic spectroscopic changes collectively support the proposed interaction based on electrostatic association between protonated ensartinib and the anionic form of eosin Y.

### Optimization reaction factors

All variables impacting the development and stability of the ENSA-eosin Y binary complex were investigated and adjusted in order to develop the assay protocols. The parameters tested include effect of temperature, eosin Y concentration, pH, and diluting solvent.

### Effect of temperature

In spectrofluorometric assays, the reaction between ENSA and the eosin reagent relies on electrostatic attraction forming an ion-pair complex, which is quantified by measuring eosin’s fluorescence quenching. The optimum temperature for this reaction is the room temperature. The strength of molecular interaction between eosin Y and ENSA was quantified through the association constant and determined using the modified Stern–Volmer equation^18,19^.

### Effect of time

Time-resolved fluorescence measurements (specifically fluorescence lifetime measurements) are an excellent tool to validate the interaction pathway of Eosin Y (EY) and ENSA, often providing more definitive evidence of interaction mechanisms than steady-state measurements alone. While steady-state spectrofluorometric measures the average intensity (quenching), time-resolved fluorescence measures the excited-state lifetime (t), which is highly sensitive to changes in the immediate environment of the dye, such as electrostatic interaction, complex formation, or changes in solvent polarity.

### Effect of pH and buffer volume

**Fig. 3:**
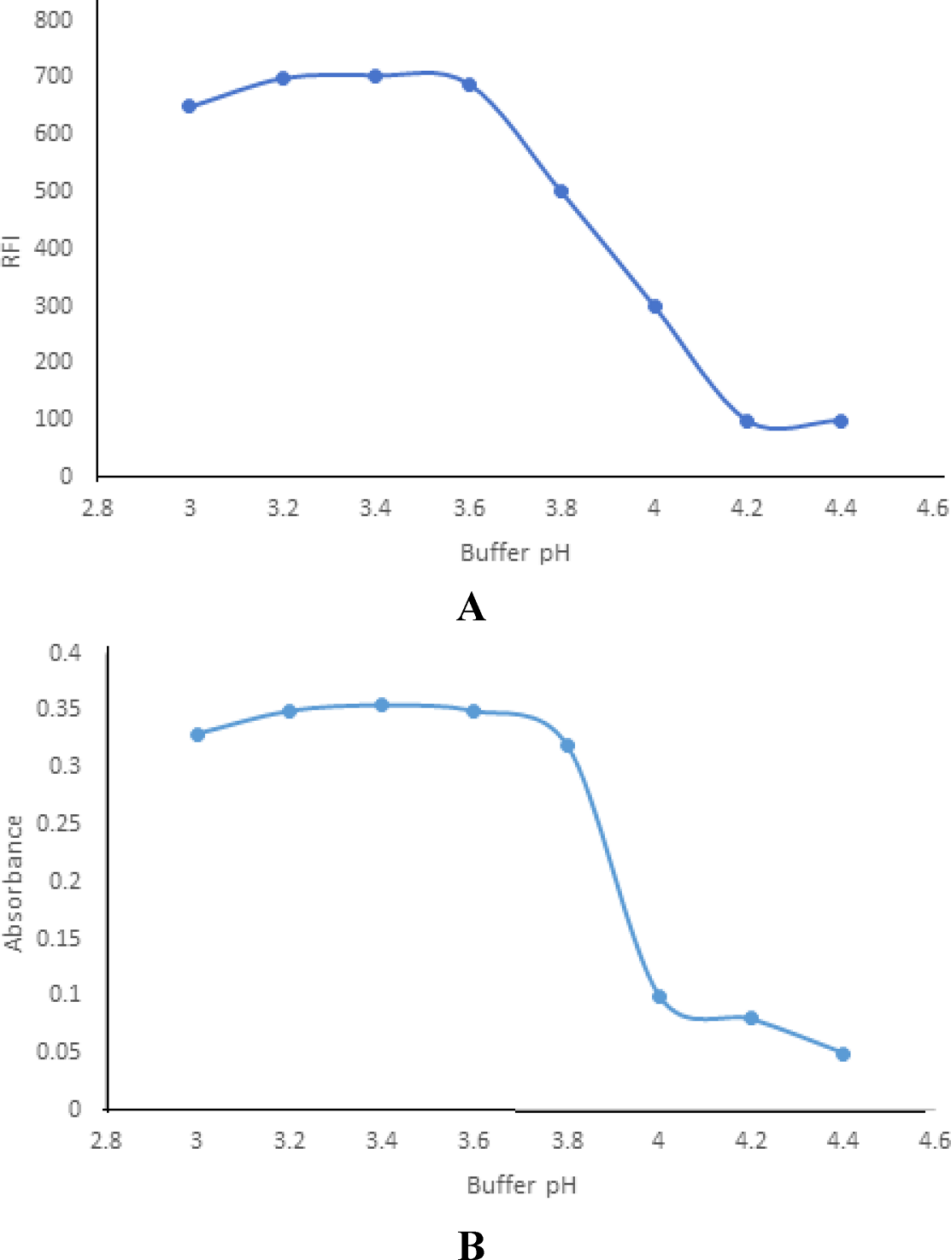
Effect of acetate buffer pH on the reaction of eosin Y (5 × 10^− 4^ M) with 300 ng mL^− 1^ ENSA for the spectrofluorometric method **(A)** and eosin Y (1 × 10^− 3^ M) with 5 µg mL^− 1^ ENSA for the spectrophotometric method **(B).**

**Fig. 4:**
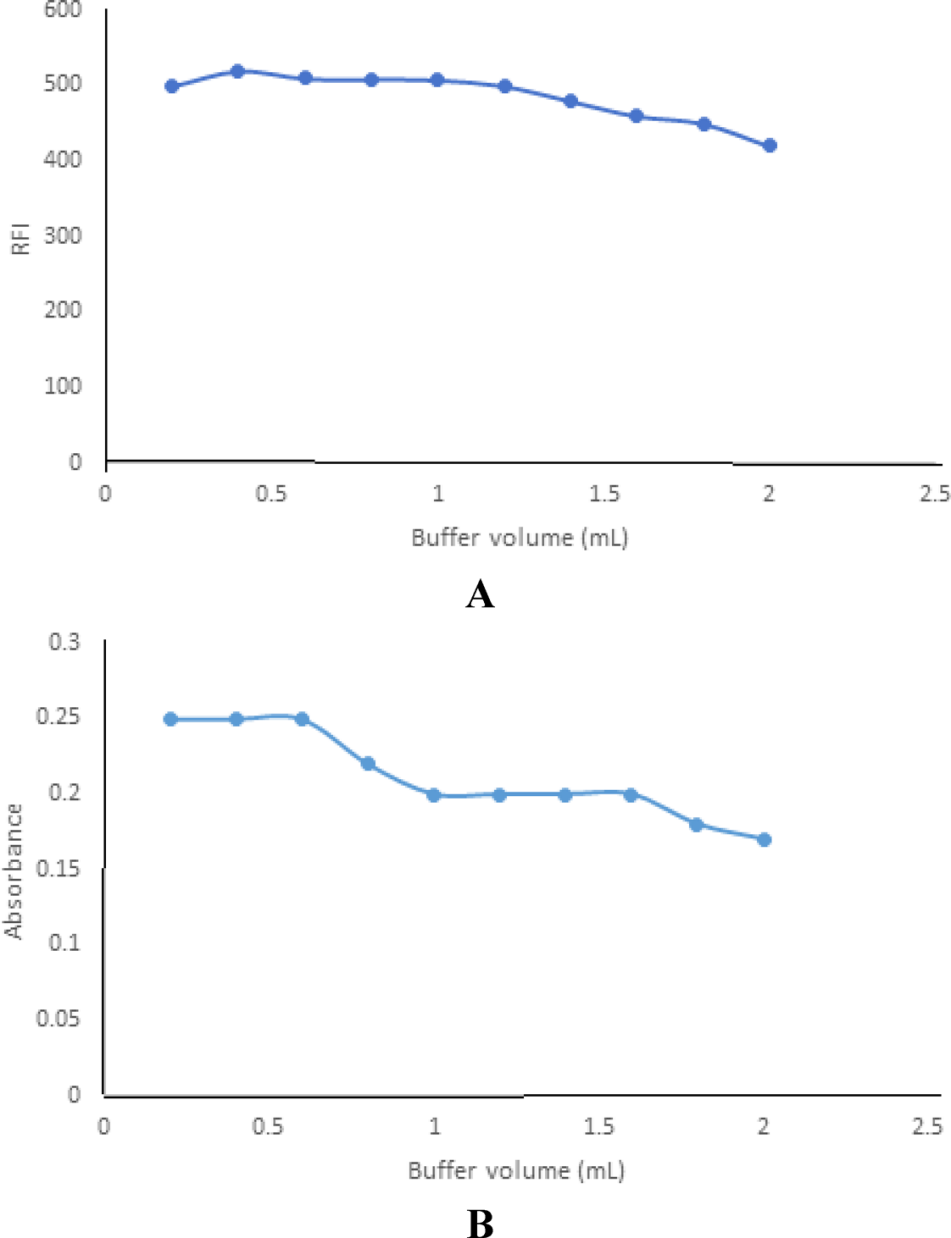
Effect of acetate buffer volume on the reaction of eosin Y (5 × 10^− 4^ M) with 300 ng mL^− 1^ ENSA for the spectrofluorometric method **(A)** and eosin Y (1 × 10^− 3^ M) with 5 µg mL^− 1^ ENSA for the spectrophotometric method **(B).**

An acidic medium was necessary to allow for efficient interaction between ENSA and eosin Y. As a result, the pH impact on complex formation was examined throughout a limit of 3.0-4.4. Maximum fluorescence quenching (Fig. 3 A) and absorbance responses (Fig. 3 B) were achieved in the pH range of 3.2–3.6, with pH 3.3 chosen for both approaches. At pH values above 3.6, the analytical response gradually decreased, which may be attributed to reduced protonation of the basic amino group(s) of ENSA. This lowers the concentration of the cationic drug species available for electrostatic interaction with anionic eosin Y, thereby decreasing ion-pair complex formation and the corresponding spectroscopic response. The study found that 1.0 ± 0.2 mL of buffer acetate (0.1 M) produced the best results for both approaches (Fig. 4 A&B).

### Impacts of the Eosin Y volume

**Fig. 5:**
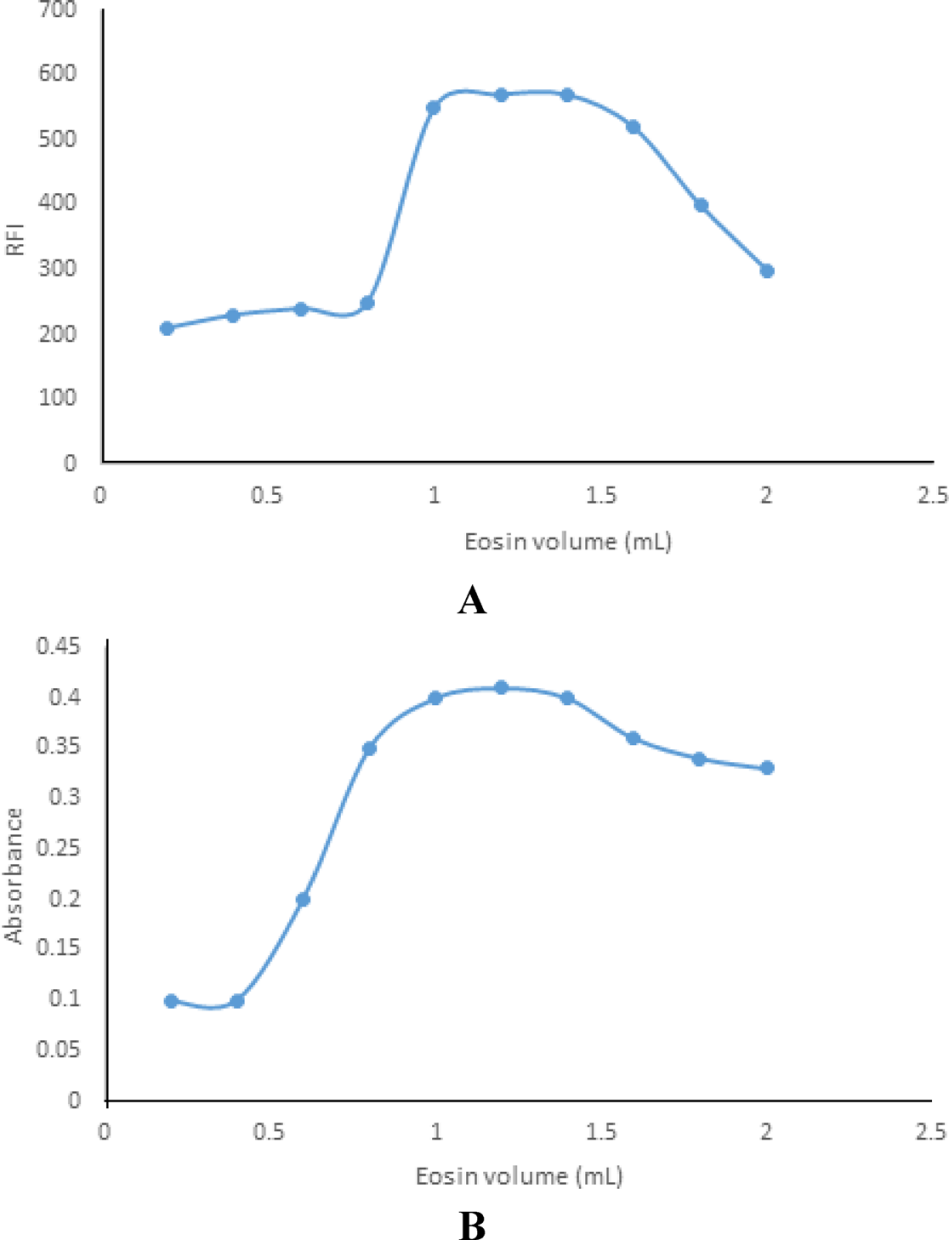
Effect of volume of eosin Y (5.0 × 10^− 4^ M) on reaction with ENSA 300 ng mL^− 1^ for spectrofluorometric method **(A)** and volume of eosin Y (1 × 10^− 3^ M) with ENSA 5 µg mL^− 1^ for spectrophotometric method **(B).**

Different volumes of eosin Y reagent were investigated while maintaining a final volume of 10 mL in calibrated volumetric flasks. The optimum analytical response was achieved using 1.3 mL of eosin Y solution (5.0 × 10 − 4 M) for Method A and 1.2 mL of eosin Y solution (1.0 × 10 − 5 M) for Method B (Figs. 5 A & 5B).

**Fig. 6:**
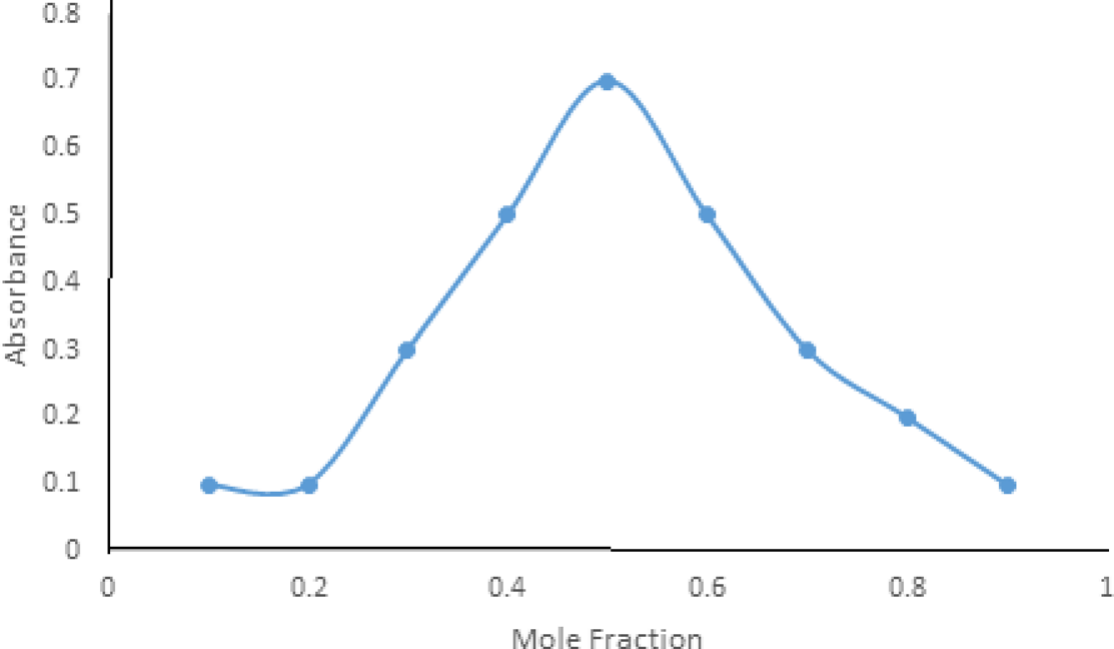
Continuous variation plot for the ion association of ENSA with eosin Y (the concentration used 1.5 × 10^− 3^ M of each).

**Fig. 7:**
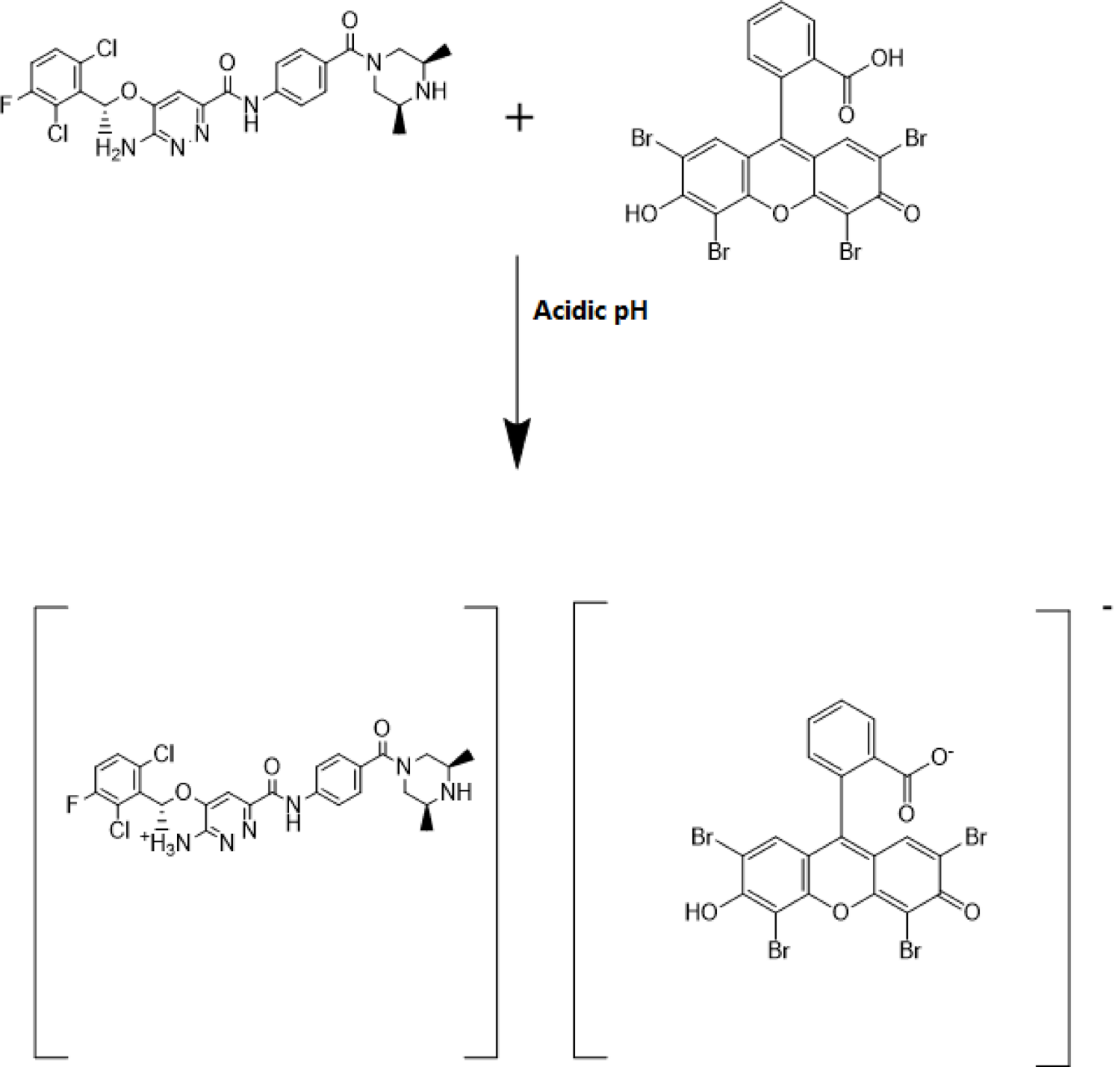
The suggested mechanism for the binary complex formation between ENSA and eosin Y.

### The dispersing liquid impact

The influence of the diluting solvent was examined using water, methanol, ethanol, acetonitrile, acetone, and dimethylformamide. Among the tested media, water produced the highest analytical response in both methods. This behavior may be attributed to efficient ionization of ENSA and eosin Y in aqueous medium, which favors ion-pair complex formation and enhances fluorescence quenching efficiency in Method A, as well as absorbance response in Method B. In addition, the high polarity of water stabilizes the charged interacting species more effectively than organic solvents. Representative spectra in different solvents are provided in the Supporting Information.

### Effect of the reaction time on ENSA–Eosin Y interaction

The influence of time to reaction on the ENSA-Eosin Y interaction was examined. Complex formation happened instantly and remained stable for at least 60 min, allowing for flexible measurement time.

### Stochiometric ratio

Job’s approach revealed a 1:1 ratio (Eosin Y: ENSA), in which one eosin Y molecule interacts with one basic amino group of ENSA to create an ion-pair complex (Fig. 6). To further clarify, Job’s method of continuous variation was performed using equimolar solutions of ENSA and eosin Y while maintaining the total molar concentration constant. The corrected absorbance values were plotted against the mole fraction of ENSA. As shown in Fig. 6, the maximum analytical response was observed at the equimolar ratio, confirming the formation of a 1:1 association complex between ENSA and eosin Y. The anticipated interaction mechanism is illustrated in Fig. 7. Based on the pH-dependent analytical behavior, the observed spectral changes, and the stoichiometric findings obtained from Job’s plot, a probable interaction pathway is proposed in Fig. 7. Under acidic conditions, protonation of the basic amino functionality in ENSA enhances its electrostatic attraction with the anionic form of eosin Y, leading to ion-pair association. The presented scheme is therefore a proposed mechanistic representation consistent with the experimental observations.

### Validation of the method

The proposed methods were validated according to ICH Q2(R1) guidelines^20^. The evaluated performance characteristics included linearity and range, sensitivity (LOD and LOQ), accuracy, precision, robustness, and applicability to pharmaceutical dosage forms. Linearity was assessed using calibration curves over the specified concentration ranges. Accuracy was evaluated through recovery studies using the standard addition procedure. Precision was examined as intra-day and inter-day repeatability at different concentration levels. Robustness was studied by applying small deliberate changes to critical experimental parameters. The long-term stability of ENSA–eosin Y complexes has been assessed, particularly in the context of validating analytical methods and determining the shelf-life of drug products (e.g., in pharmaceutical ion-pair complexes) and in the study of aging for eosin Y-based complexes.

### Linearity and range

Excellent linearity was observed over the range of concentration 100–1000 ng/mL for Method A and 1–10 µg /mL for Method B. The correlation coefficients were 0.9996 and 0.9998, respectively (Table 1), confirming the suitability of the calibration curves for quantitative analysis.

**Table 1:** The regression and validation parameters for the proposed spectroscopic methods for the determination of ENSA.

Parameter	Spectrofluorometric method (Method A)	Spectrophotometric method (Method B)
Linearity range (ng mL^− 1^)	100–1000	1000–10,000
Slope	2.46	0.100
SD of slope	0.023	0.001
Intercept	761.64	− 0.004
SD of intercept	15.77	0.004
Correlation Coefficients	0.9996	0.9998
LOD (ng mL^− 1^)	21.15	132.0
LOQ (ng mL^− 1^)	64.11	400.0

### Detection and quantitation limits

Limits of detection and quantification were determined according to ICH equations using the standard deviation of the intercept and the slope of the calibration curve. LOQ values were 64.11 ng mL⁻¹ for Method A and 400.0 ng mL⁻¹ for Method B, while LOD values were 21.15 ng mL⁻¹ and 132.0 ng mL⁻¹, respectively. Although chromatographic LC–MS/MS methods may achieve lower detection limits, the sensitivity of the proposed spectroscopic methods is sufficient for routine pharmaceutical analysis while offering advantages of operational simplicity, rapidity, reduced cost, and environmentally friendly conditions. The limits of detection and quantification were calculated using the following equations: LOD = 3.3 × (σ / S) and LOQ = 10 × (σ / S).

Where σ is the standard deviation of intercept and S is the slope.

### Precision and accuracy

Accuracy was evaluated using recovery studies at three concentration levels of ensartinib (low, medium, high), each analyzed in triplicate. The recovery values ranged between 98.51% and 101.57%, demonstrating excellent agreement with the true values. Precision was assessed as intra‑day and inter‑day repeatability. For inter‑day precision, the same procedure was repeated on three consecutive days, while intra‑day precision, the same procedure was repeated on the same day. The relative standard deviation (RSD) values were consistently below 2%, confirming the high reproducibility of the proposed methods. Statistical evaluation using Student’s t‑test and F‑test revealed no significant differences between intra‑ and inter‑day results at the 95% confidence level, further supporting the robustness of the methods. These findings indicate that the spectrophotometric and spectrofluorometric procedures provide reliable quantification of ensartinib in pharmaceutical dosage forms, with minimal variability across different days and at the same day. The detailed results are summarized in Tables 2 and 3, while the narrative discussion emphasizes the robustness and consistency of the methods.

**Table 2:** Accuracy of the proposed spectroscopic methods for the determination ENSA using the standard addition method.

Method	Amount, Taken (ng mL^− 1^)	Amount, Added (ng mL^− 1^)	% Recovery ± SD^a^
Method A	500	0	98.89 ± 1.32
	500	500	100.64 ± 1.08
	500	600	101.68 ± 0.99
	500	700	100.97 ± 1.45
Method B	5000	0	99.89 ± 1.111
	5000	5000	100.47 ± 0.89
	5000	7000	98.51 ± 1.68
	5000	9000	101.57 ± 1.78

^a^ Mean of three determinations.

**Table 3:** Evaluation of the intra-day and inter-day precision of the proposed spectroscopic methods for the determination of ENSA.

Method	Conc. Level (ng mL^− 1^)	% Recovery ± RSD ^a^	
		Intra-day precision	Inter day precision
Method A	300	99.52 ± 1.98	100.65 ± 0.98
	500	100.97 ± 0.65	99.46 ± 1.86
	700	101.75 ± 1.53	100.64 ± 0.78
	900	100.65 ± 0.76	99.54 ± 1.76
Method B	3000	98.64 ± 1.76	100.7 ± 0.98
	5000	99.99 ± 1.68	98.47 ± 1.67
	7000	101.54 ± 1.99	101.75 ± 0.75
	9000	98.64 ± 0.98	98.55 ± 1.85

^a^ Mean of three determinations.

### The system’s robustness testing

The robustness of the proposed methods was evaluated by introducing small deliberate variations in critical parameters, including acetate buffer pH (3.3 ± 0.1), buffer volume (± 0.1 mL), and eosin Y reagent volume (± 0.1 mL) (Table 4). The recovery values remained close to 100% with relative standard deviation values below 2%, indicating that these minor changes did not significantly affect analytical performance. This demonstrates that the methods are sufficiently robust and can maintain consistent accuracy and precision under normal laboratory conditions, even when slight variations occur during routine practice.

**Table 4:** Robustness of the proposed spectroscopic methods for the determination of ENSA.

Parameter		% Recovery ± SD^a^	
		Method A	Method B
Buffer pH, 3.3	− 0.1	100.76 ± 1.75	99.36 ± 1.65
	+ 0.1	99.75 ± 0.95	100.86 ± 0.97
Buffer volume, 1 mL	− 0.1 mL	101.86 ± 1.24	100.75 ± 1.54
	+ 0.1 mL	100.42 ± 1.86	99.74 ± 1.11
Eosin volume, 1.2 mL	− 0.1 mL	98.65 ± 1.44	102.75 ± 0.53
	+ 0.1 mL	101.29 ± 0.89	100.33 ± 1.75

^a^ The values are the mean of three determinations.

### Evaluations of available prescribed formulations

The described methodologies were successfully applied to the determination of ENSA in commercial capsule dosage forms. The obtained recovery values were close to 100%, demonstrating satisfactory accuracy of the proposed methods in the presence of common formulation excipients. The low standard deviation values further confirm good precision and repeatability during routine analysis. A statistical comparison with a previously published chromatographic method^21^ using Student’s t-test and F-test indicated no significant difference at the 95% confidence level, confirming the reliability of the proposed procedures. Although chromatographic methods may provide higher instrumental sensitivity, the present methods offer practical advantages for routine quality-control laboratories, including operational simplicity, rapid analysis, lower cost, and no requirement for sophisticated instrumentation or extensive sample pretreatment (Table 5).

**Table 5:** Application of the proposed spectroscopic method for the determination of ENSA in dosage form.

parameters	Reported method^7^	Method A	Method B
% Recovery ^a^	99.89	99.29	100.39
Standard deviation, ±SD	1.23	1.11	0.98
Number of determinations	5	3	3
t – value ^a^		0.710	0.634
F – value ^a^		1.228	1.575

^a^ Tabulated value at 95% confidence limit; t = 2.365 and F = 5.41.

**Table 6:**
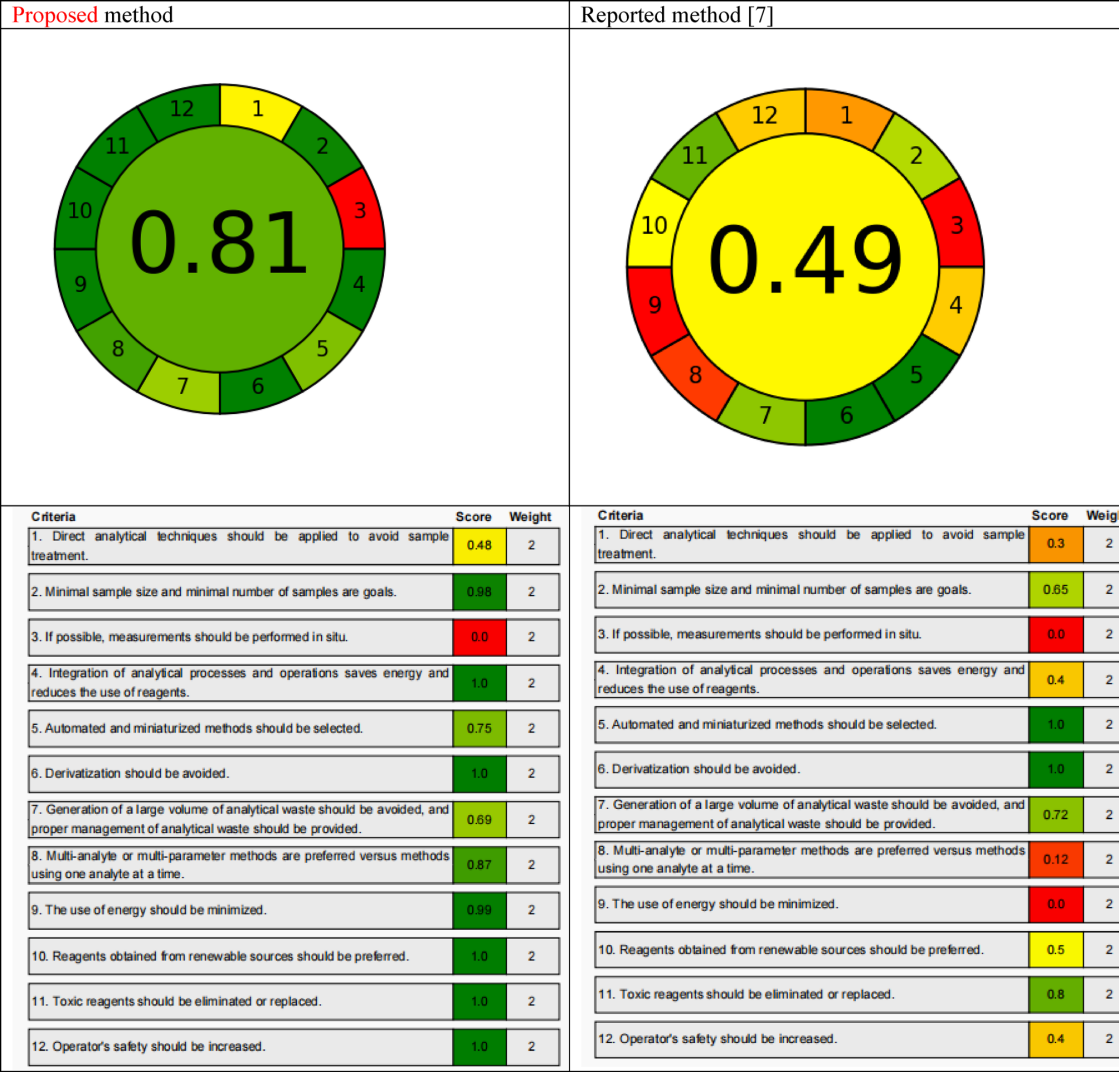
Greenness evaluation of the proposed & reported method using AGREE.

### System greenness assessment

The ecological effect of the analytical procedures was evaluated using the AGREE and GAPI tools^17–20^. In the GAPI pictogram, the green, yellow, and red fields represent low, moderate, and high environmental impact, respectively, for each stage of the analytical workflow, including sample preparation, reagent consumption, solvent type, instrumentation, and waste generation. This visualization clearly demonstrates the minimal environmental burden of the proposed methods. Due to the use of water as the main diluting solvent, the absence of complex sample pretreatment, and the avoidance of hazardous organic solvents, GAPI assessment indicated acceptable environmental performance. In the AGREE diagram, the circular segmented layout corresponds to the twelve principles of Green Analytical Chemistry, where the color gradient from red to green reflects increasing compliance with each individual principle. According to the AGREE evaluation scale, scores below 0.40 indicate low greenness, values between 0.40 and 0.60 represent moderate compliance, while scores ranging from 0.60 to 0.80 reflect high greenness, and values above 0.80 indicate excellent adherence to green analytical chemistry principles. Accordingly, the obtained AGREE score of approximately 0.81 classifies the proposed methods as an excellent green analytical procedure. The environmental impact of the proposed spectroscopic methods was assessed using the GAPI and AGREE tools, and the results were compared with those of a published chromatographic reference method for ensartinib determination in pharmaceutical dosage forms (Table 6). The spectroscopic procedures achieved an AGREE score of 0.81, classifying them as excellent green methods, while the reference chromatographic method showed lower greenness due to extensive use of organic solvents and complex sample preparation. In addition, whiteness evaluation, which integrates greenness with analytical performance and practical applicability, confirmed the superiority of the proposed methods. The spectroscopic procedures demonstrated high whiteness scores, reflecting their balance between environmental sustainability, sensitivity, accuracy, and routine applicability. In contrast, the reference method, although highly sensitive, exhibited lower whiteness due to reduced environmental compatibility and higher operational complexity. These findings highlight that the developed spectrophotometric and spectrofluorometric methods not only provide reliable analytical performance but also represent greener and whiter alternatives to conventional chromatographic techniques for routine quality‑control analysis of ensartinib.

## Conclusion

The present study introduces the first spectrophotometric and spectrofluorometric methods for the determination of ensartinib in pharmaceutical dosage forms. The methods are characterized by simplicity, rapidity, cost‑effectiveness, and environmental compatibility, relying on aqueous media and avoiding hazardous solvents. Validation studies confirmed excellent accuracy, precision, and robustness, with recovery values close to 100% and relative standard deviations below 2%. The limits of detection and quantification, although higher than those reported for LC–MS/MS assays, are adequate for routine dosage‑form analysis. Statistical comparison with a reference chromatographic method revealed no significant differences at the 95% confidence level, confirming the reliability of the proposed procedures. Greenness and whiteness evaluations further demonstrated the superiority of the methods over conventional chromatographic techniques. These findings highlight the novelty and practical applicability of the developed spectroscopic approaches, offering sustainable alternatives for routine quality‑control laboratories.

## Data Availability

The datasets used and/or analysed during the current study available from the corresponding author on reasonable request.
